# Exploring Compound Eyes in Adults of Four Coleopteran Species Using Synchrotron X-ray Phase-Contrast Microtomography (SR-PhC Micro-CT)

**DOI:** 10.3390/life12050741

**Published:** 2022-05-17

**Authors:** Anita Giglio, Maria Luigia Vommaro, Raffaele Giuseppe Agostino, Lai Ka Lo, Sandro Donato

**Affiliations:** 1 Department of Biology, Ecology and Earth Science, University of Calabria, Via Bucci, Arcavacata di Rende, 87036 Cosenza, Italy; marialuigia.vommaro@unical.it; 2 Department of Physics and STAR-LAB, University of Calabria, Via Bucci, Arcavacata di Rende, 87036 Cosenza, Italy; raffaele.agostino@fis.unical.it (R.G.A.); sandro.donato@fis.unical.it (S.D.); 3 Consiglio Nazionale delle Ricerche, Istituto di Nanotecnologia (Nanotec)—UoS Cosenza, Via Bucci, Arcavacata di Rende, 87036 Cosenza, Italy; 4 Animal Evolutionary Ecology Group, Institute for Evolution and Biodiversity, University of Münster, 48149 Münster, Germany; lo@uni-muenster.de; 5 Istituto Nazionale di Fisica Nucleare, Division of Frascati, Via Fermi, 54, Frascati, 00044 Rome, Italy

**Keywords:** beetle, brain, cornea, microtomography, morphology, ommatidia, optical lobe, rendering, virtual sectioning, visual system

## Abstract

Compound eyes in insects are primary visual receptors of surrounding environments. They show considerable design variations, from the apposition vision of most day-active species to the superposition vision of nocturnal insects, that sacrifice resolution to increase sensitivity and are able to overcome the challenges of vision during lightless hours or in dim habitats. In this study, Synchrotron radiation X-ray phase-contrast microtomography was used to describe the eye structure of four coleopteran species, showing species-specific habitat demands and different feeding habits, namely the saproxylic *Clinidium canaliculatum* (Costa, 1839) (Rhysodidae), the omnivorous *Tenebrio molitor* (Linnaeus, 1758) and *Tribolium castaneum* (Herbest, 1797) (Tenebrionidae), and the generalist predator *Pterostichus melas italicus* (Dejean, 1828) (Carabidae). Virtual sections and 3D volume renderings of the heads were performed to evaluate the application and limitations of this technique for studying the internal dioptrical and sensorial parts of eyes, and to avoid time-consuming methods such as ultrastructural analyses and classic histology. Morphological parameters such as the area of the corneal facet lens and cornea, interocular distance, facet density and corneal lens thickness were measured, and differences among the studied species were discussed concerning the differences in lifestyle and habitat preferences making different demands on the visual system. Our imaging results provide, for the first time, morphological descriptions of the compound eyes in these species, supplementing their ecological and behavioural traits.

## 1. Introduction

The application and advantages of microtomography (micro-CT) in entomology provide a significant improvement step for collecting data on the insect anatomy. This method avoids artefacts resulting from invasive dissections, followed by relatively time-consuming fixing and physical tissue slicing, required for image analyses under light and electron microscopy. Indeed, micro-CT has proven to be useful for virtual dissections, 3D reconstruction and morphological descriptions of the head [1,2], muscles [2,3,4], brain [5], digestive [6,7] and reproductive [8,9,10,11] systems, as well as insect fossils [12,13,14,15]. Moreover, Synchrotron radiation X-ray phase-contrast microtomography (SR-PhC micro-CT) allows the use of high-resolution imaging coupled with segmentation, for 3D morphological analyses with high image contrast-to-noise ratios in biological tissues, and does not require the use of contrast agents, even in samples with weak X-ray absorption [16,17,18,19]. In recent decades, it has been applied as a non-invasive technique to observe external and internal anatomical structures of living insects [20,21], and specimens immersed in ethanol after fixation [2,6] or embedded in amber [22].

In insects, compound eyes, which are paired structures located on the left and right sides of the head, contain a species-specific number of light-sensitive units named ommatidia [23,24]. Each ommatidium consists of two main components: a lens unit (consisting of an external corneal facet and a crystalline cone lens), which collects and focusses incoming light, and the rhabdom, which absorbs and transduces focussed light. The quantity of light available and the balance between resolution and sensitivity are crucial factors that define the structure and size of compound eyes, as well as their spatial resolving power [25]. The large variety of ecological niches occupied by insects explains the variability of the eye structure, which differs greatly in different visual tasks (detecting food, predator and partner recognition) across habitats; therefore, the selected eye design should reflect the lifestyle and behaviour of each species [26,27,28,29,30]. For example, visual hunters [27,31] and flying insects [32,33,34] have large compound eyes, advantageous in the search for food and partners, while species living in low light conditions show a reduction in the number of ommatidia, as observed in cave-adapted species belonging to Carabidae [35], Leiodidae [36] and Curculionidae [37]. 

Light and electron microscopy techniques have been largely applied to define the structure and function of insects’ eyes [24,38,39,40], as well as the selective pressures that impact acuity from ecological and evolutionary perspectives [41]. X-ray tomographic images of insects’ eyes have been reported in Ephemeroptera [42] or as secondary information in analyses focusing on the head structure [1,43], brain anatomy [5,44,45,46] or general anatomy of miniature insects [7]. However, there is a lack of studies applying this technique to analyse the morphological variations of compound eyes. Thus, the aim of this study was to indicate a new application of SR-PhC micro-CT for investigating compound eyes in insects. Virtual sections and 3D renderings of the head were performed in four coleopteran species, inhabiting different habitats and with different ecological roles, i.e., (a) *Clinidium canaliculatum* (Costa, 1839) (Rhysodidae), a saproxylic beetle, which feeds on wood-decomposing fungi in coniferous forests—listed as a vulnerable species in the red list of the International Union for Conservation of Nature (IUCN) [47,48]; (b) *Tenebrio molitor* (Linnaeus, 1758) and *Tribolium castaneum* (Herbst, 1797) (Tenebrionidae), pests of stored grain and cosmopolitan in distribution [49]; and (c) *Pterostichus melas italicus* (Dejean, 1828) (Carabidae), a generalist predator, inhabiting pastures, open forests, forest edges and agricultural land [50], well known as a bioindicator of exposure to agrochemicals [51,52,53]. The study was designed to provide a proof that high-resolution images of compound eyes can be obtained using SR-PhC micro-CT as an exploratory alternative to invasive and time-consuming techniques. To the best of authors’ knowledge, this is the first comparative study on insect compound eyes using this technique and addresses the lack of information in the literature on the eyes of the investigated species.

## 2. Materials and Methods

### 2.1. Insects

*Clinidium canaliculatum* specimens were hand-collected under rotten pine bark in the Sila National Park (39°21′16.79″ N, 16°37′57.64″ E, Monte Spina 1550 m a.s.l., San Giovanni in Fiore, Calabria, Southern Italy) in May 2021. Adults of *P. m. italicus* were collected from their natural habitat in an olive grove (39°59′07.56″ N, 16°15′32.64″ E, 1202 m a.s.l., San Marco Argentano, Calabria, Southern Italy) using pitfall traps (plastic jars 200 mL in volume containing fruit as an attractant), in October 2019. In the laboratory, beetles of both species were identified by using dichotomous keys [54] and separated by gender. 

*Tenebrio molitor* specimens were obtained from a laboratory stock population maintained at the Morphofunctional Entomology Laboratory, Dept. of Biology, Ecology and Earth Science, University of Calabria. Mealworm beetles were reared at 60% relative humidity, under a natural photoperiod and room temperature (23 ± 2 °C), with an *ad libitum* diet of organic wheat meal and fruit. 

Specimens of *T. castaneum,* belonging to the strain Croatia 1 (CRO1), were collected and isolated from a wild population in Croatia [55], and reared under laboratory conditions over generations. Adult beetles, kept in plastic boxes, were fed with heat-sterilised (75 °C for at least 24 h) organic wheat flour with 5% brewer’s yeast powder, and reared at 30 °C, 70% humidity and with a 12:12 h light:dark cycle.

### 2.2. Sample Preparation

Males and females from each species were anaesthetised in a cold chamber at 4 °C for three minutes and prepared as indicated in [6]. Briefly, beetles were fixed in 2.5% glutaraldehyde and 1% paraformaldehyde in 0.1 M phosphate buffer, at pH 7.4 (PBS; Electron Microscopy Sciences), overnight at 4 °C, washed with PBS and dehydrated in a graded ethanol series. The following number of individuals was used for each species: one male and one female for *C. canaliculatum* and *P. m. italicus,* and 2 males and 2 females for *T. molitor* and *T. castaneum.*

### 2.3. Phase-Contrast Micro-Computed Tomography (PhC micro-CT) and Data Acquisition

To digitally reconstruct the three-dimensional internal anatomy of beetles, we used a Synchrotron radiation X-ray phase-contrast micro-computed tomographic (SR-PhC micro-CT) imaging technique. Tomographic acquisitions were performed at the SYRMEP beamline of the Elettra synchrotron facility in Trieste (Italy), in the “white-beam” configuration mode, i.e., illuminating the sample with polychromatic X-ray radiation [56,57]. A storage ring-bending magnet produces the X-ray beam, available at the beamline in the energy range from 8.5 to 40 keV. To compensate for beam hardening effects, we filtered the X-ray beam for low energy components using 1.0 mm of Silicon, thus resulting in an average energy of around 20 keV. Considering the natural divergence of the X-rays produced by the source, the beam cross-section at the sample position (22.5 m away from the source) is 150 mm (horizontal) × 5 mm (vertical). The imaging system consisted of a water cooled Hamamatsu sCMOS detector (with sensors providing 2048 × 2048 pixels each, with a size of 6.5 µm × 6.5 µm), coupled optically with a GGG (Gd3Ga5O12:Eu) scintillator, and utilising a set of optical lenses that enabled the setting of different magnification levels. 

We employed the GGG scintillator with a 17 µm thick sensitive layer to acquire images of *C. canaliculatum* and *T. castaneum*, while for *P. m. italicus* and *T. molitor,* we used a GGG with a sensitive layer with a thickness of 45 µm. Tomographic images were reconstructed from 1800 evenly spaced projections, spanning over 180 degrees, and collected in continuous rotation mode. Projection images were obtained in the propagation-based phase-contrast regime [18,58], setting a propagation distance between the sample and the detector. The propagation distance was set to optimise the signal-to-noise ratio in the near-field regime, once the pixel size had been set [59]. Phase-contrast effects emerging from the free-space propagation result in an enhanced contrast arising at the boundaries between details with different compositions (the so-called edge-enhancement). For *P. melas italicus*, the optical magnification was set to 2.4, resulting in a pixel size of 2.7 µm × 2.7 µm and a lateral field of view of 5.5 mm × 5.5 mm. The exposure time was set to 250 ms/projection and the sample-to-detector distance was 150 mm. Four vertical scans were needed to image the full length of the sample. For *T. molitor*, the optical magnification was set to 1.6, resulting in a pixel size of 4.0 µm × 4.0 µm and a lateral field of view of nearly 8.1 mm × 8.1 mm. The exposure time was set to 150 ms/projection and the sample-to-detector distance was 250 mm. Four vertical scans were needed to image the full length of the sample.

For *C. canaliculatum* and *T. castaneum*, the optical magnification was set to 4.3, resulting in a pixel size of 1.5 µm × 1.5 µm and a lateral field of view of nearly 3.1 mm × 3.1 mm. The exposure time was set to 200 ms/projection and the sample-to-detector distance was 100 mm. Four and two vertical scans were needed to image the full length of *C. canaliculatum* and *T. castaneum*, respectively.

### 2.4. Computer-Based 3D Reconstruction and Segmentation

Image reconstruction was performed with a GPU-based filtered back-projection algorithm (applying a Shepp–Logan filter), using the SYRMEP Tomo Project (STP) software suite [60]. Before image reconstruction, projections were further processed using a phase-retrieval filter, based on the homogeneous transport of intensity equation (TIE-Hom) [61], obtaining a higher signal-to-noise ratio at the cost of a loss of edge-enhancement signal [62]. The filter parameter, δ/β, was tuned to effectively regulate the amount of smoothing, as usually used in experimental practice. For the four specimens, we set δ/β = 400. After processing, the final CT reconstruction yields a 3D map which is substantially proportional to the linear attenuation coefficient of the sample [63,64]. Volume renderings of different sections of the beetles were performed using the scientific visualisation software Drishti [65] and Avizo^®^ 3D. 

### 2.5. Image Analyses and Measurements

**Figure 1:**
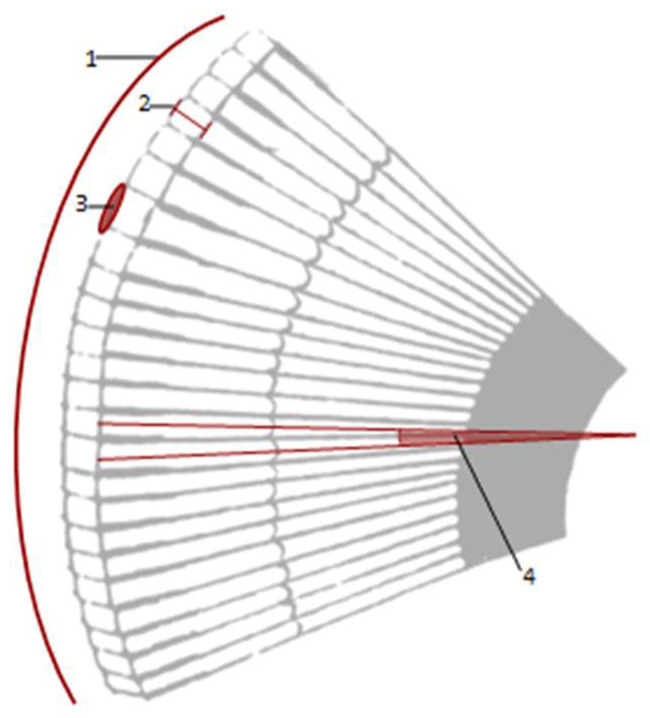
Drawing shows morphometric measurements on compound eye: **1** corneal surface, **2** corneal facet lens thickness **3** corneal facet surface area and **4** interommatidial angle.

Morphometric measurements on 2D virtual slices were assessed with the open-source software ImageJ [66] on digitised images and processed as mean ± standard deviation. For each species, the following measurements were taken: the area of the corneal facet lens and cornea, interocular distance, facet density and corneal lens thickness (Figure 1). To define the differences in the total surface between the cornea and ommatidia facet lenses, the measures were also performed on the segmentation of the lens by using the “Generate Surface” and “Surface Area/Volume” modules of the Avizo software. The area of the corneal facet lens surface was measured as πd^2^/4 (d = diameter of facet). The interocular distance, calculated on the volume rendering of the head, was measured as the frontal distance between the inner edges of both eyes, at the level of the central row of the ommatidium. The facet density (mm^−2^) was calculated as the ratio between the number of ommatidia (n) and the surface area of the cornea. The ommatidial axis was taken as a line through the midpoint of the rhabdom and the corneal lens, and the interommatidial angle was measured from line drawings by two continuous ommatidia on 2D virtual sections.

## 3. Results

**Figure 2:**
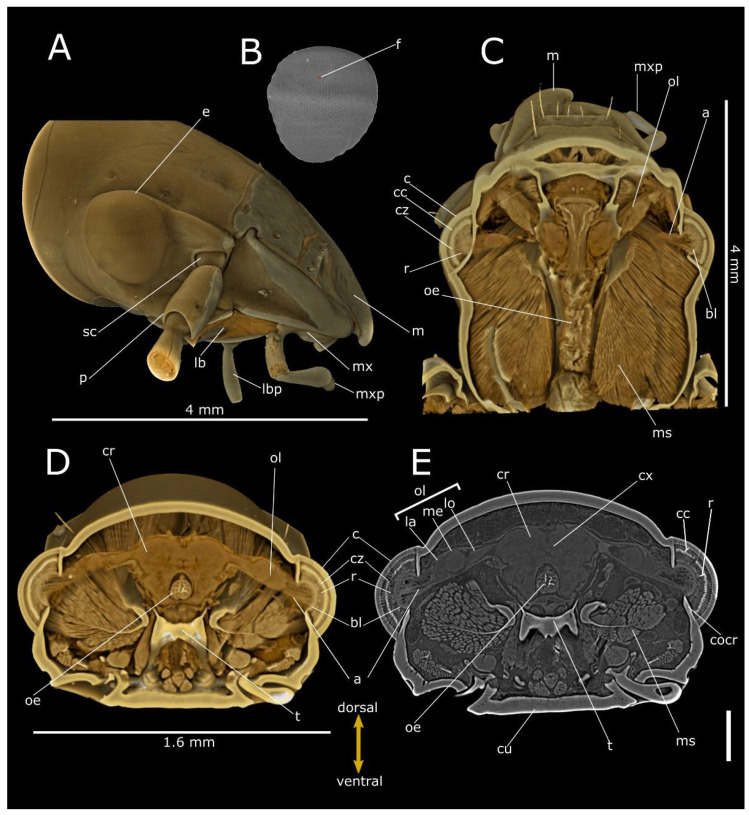
Phase-contrast micro-CT analysis of *Pterostichus melas italicus* head. Volume renderings of lateral view (**A**), segmented corneal (**B**), frontal (dorsal view) (**C**) and cross (**D**) sections. Virtual 2D cross section (**E**) showing the compound eyes connecting to the cerebrum (cr) through the optical lobe (ol). a: axones; bl: basal lamina; c: cornea; cu: cuticle; cx: central complex; cc: crystalline cones; cz: clear zone; cocr: circumocular ridge; e: compound eye; f: facet; la: lamina; lb: labium; lbp: labial palp; lo: lobula; m: mandible; me: medulla; ms: muscles; mx: maxilla; mxp: maxillary palp; oe: oesophagus; p: pedicellum; r: rhabdoms; sc: scape; t: tentorial bridge. Bar: 500 µm.

**Figure 3:**
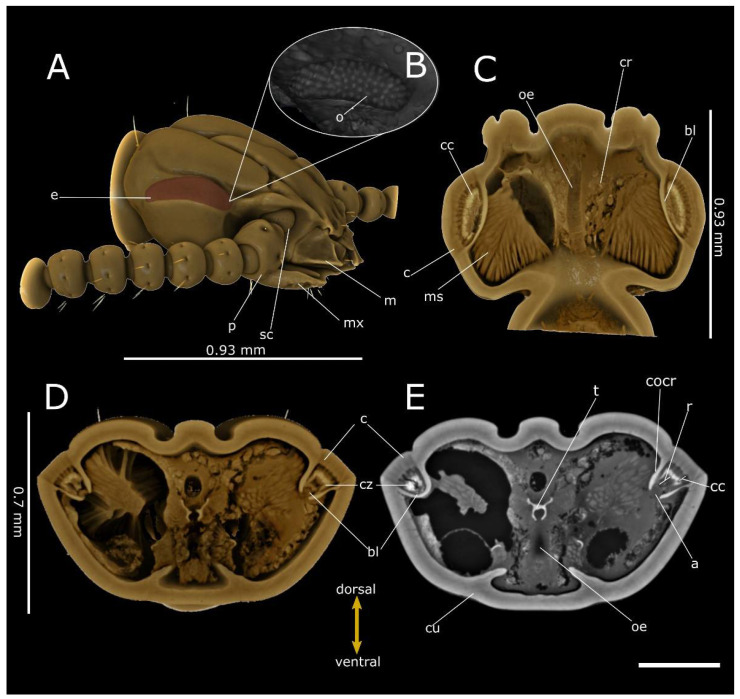
Phase-contrast micro-CT analysis of *C. canaliculatum* head. Volume renderings of the head. The lateral view (**A**) shows the flattened area of the cornea (brown) covering the ommatidia, which are visible through the virtual cuticle removal (**B**). Frontal (dorsal view) (**C**) and cross (**D**) sections highlight compound eyes’ internal structures. Virtual 2D slice of the cross-section (**E**) showing the compound eyes connecting to the cerebrum (cr) through the optical lobe (ol). a: axones; bl: basal lamina; c: cornea; cu: cuticle; cc: crystalline cones; cocr: circumocular ridge; cz: clear zone; e: compound eye; m: mandible; ms: muscles; mx: maxilla; o: ommatidium; oe: oesophagus; p: pedicellum; r: rhabdoms; sc: scape; t: tentorium. Bar: 250 µm.

**Figure 4:**
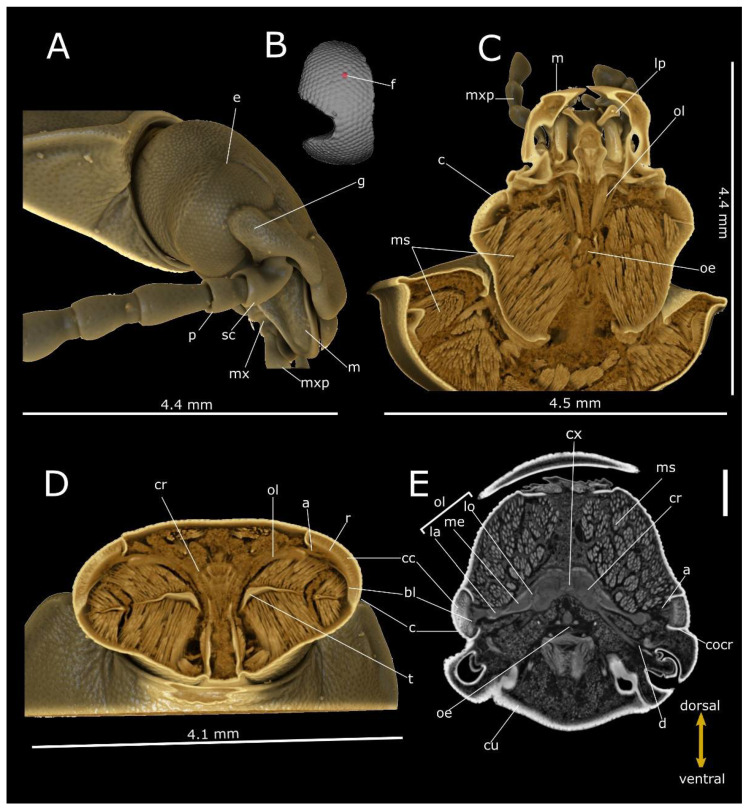
Phase-contrast micro-CT analysis of *T. molitor* head. Volume renderings of head showing lateral view (**A**), segmented cornea (**B**), frontal (dorsal view) (**C**) and cross (**D**) sections. (**E**) Virtual 2D slice of the cross-section showing the compound eyes connecting to the cerebrum (cr) through the optical lobe (ol). a: axones; bl: basal lamina; c: cornea; cu: cuticle; cx: central complex of cerebrum; cc: crystalline cones; cocr: circumocular ridge; d: deuterocerebrum; e: compound eye; f: facet; g: gena; la: lamina; lb: labium; lo: lobula; m: mandible; me: medulla; ms: muscles; mx: maxilla; mxp: maxillary palp; oe: oesophagus; p: pedicellum; r: rhabdoms; sc: scape; t: tentorium. Bar: 500 µm.

**Figure 5:**
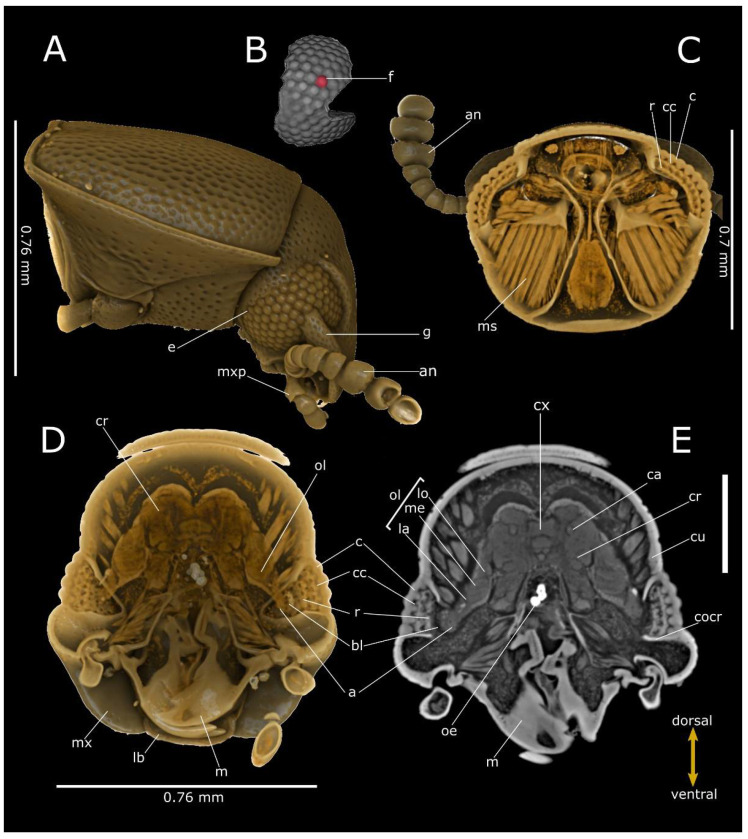
Phase-contrast micro-CT analysis of *T. castaneum* head. Volume renderings of head showing lateral view (**A**), segmented cornea (**B**), frontal (dorsal view) (**C**) and cross (**D**) sections. Virtual 2D cross-section (**E**), showing the compound eyes connecting to the cerebrum (cr) through the optical lobe (ol). a: axones; an: antenna; bl: basal lamina; c: cornea; cu: cuticle; cb: central body; ca: calyx; cc: crystalline cones; cocr: circumocular ridge; e: compound eye; f: facet; g: gena; la: lamina; lb: labrum; lp: labial palp; lo: lobula; m: mandible; me: medulla; ms: muscles; mx: maxilla; mxp: maxillary palp; oe: oesophagus; r: rhabdoms. Bar: 250 µm.

**Figure 6:**
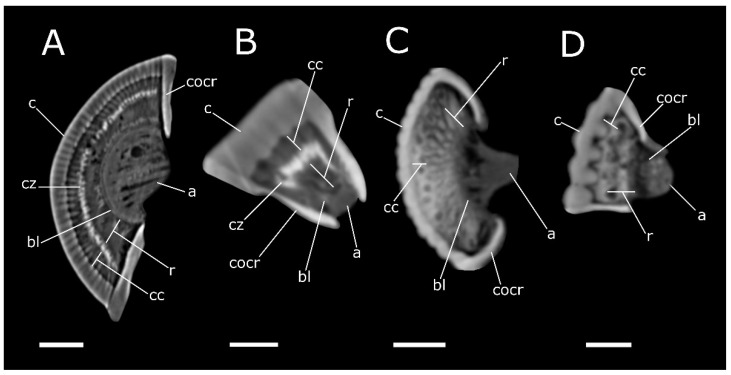
Phase-contrast micro-CT analysis, virtual 2D cross-sections of compound eyes in *P. melas* (**A**), *C. canaliculatum* (**B**), *T. molitor* (**C**) and *T. castaneum* (**D**). a: axones; bl: basal lamina; c: cornea; cc: crystalline cones; cocr: circumocular ridge; cz: clear zone; r: rhabdoms. Bar: 150 µm (**A**,**C**), 50 µm (**B**,**D**).

The complete series of virtual sections and 3D reconstructions of the heads for each analysed species allowed us to describe the external morphology of the eyes and their internal dioptrical and sensorial parts (Figure 2, Figure 3, Figure 4, Figure 5 and Figure 6).

The compound eye of *P. melas* (Figure 2A–E and Figure 6A) has a hemispherical curved area of 6.9 × 10^5^ µm^2^ and a 2000 n/mm^2^ density of facets (Table 1; Figure 2A,B).

The adjacent ommatidia are covered by a regular biconvex corneal facet lens, having a thickness of 64.9 ± 6.07 µm (N = 13), while the interocular distance was estimated at approximately 2.73 mm (Table 1). We estimated approximatively 1380 ommatidia. Virtual sections (Figure 2E and Figure 6A) and 3D reconstructions (Figure 2C–D) highlight the clear zone, characterised by a high level of X-ray attenuation (bright pixels), between the upper crystalline cone layer and the underlying layer (rhabdom), both of which have lower attenuation. The dioptric apparatus is covered by the basal lamina. The axons are connected to the optic lobe, clearly distinguishable from the distal part in the lamina, medulla and lobula, connected to the cerebrum (Figure 2C–E). The interommatidial angle was 4.09 ± 0.66° (N = 13).

The volume renderings and virtual sections of the *C. canaliculatum* head (Figure 3A–E) highlight an ocular elliptic flattened area of 4.5 × 10^4^ µm^2^ and a measured thickness of 50.94 ± 2.73 µm (N = 19) (Table 1). The cornea is smooth and the external facets of the corneal lens marking the position of the ommatidia are indistinguishable in both males and females. However, the virtual cuticle removal from the head shows a cluster of 70 ommatidia (Figure 3B), corresponding to the area of (2.09 ± 0.56) × 10^4^ µm^2^ (N = 15), which is smaller than the surface area of the cornea, revealing a facet density of 1555 n/mm^2^. Moreover, crystalline cones and rhabdoms show low attenuation if compared to the intermediate clear zone (retina), which is clearly defined by the difference in attenuation (Figure 6B). Rhabdoms are lined by the basal lamina and axons are visible in the virtual renderings and 2D sections of the eyes (Figure 3C–E), connecting with the cerebrum. The interommatidial angle was 7.36 ± 1.25° (N = 8).

The compound eyes of both tenebrionid species are dorsoventral extended and cover a large part of the lateral head. They exhibit a characteristic bilobed shape, due to a protrusion of the strongly expanded gena in the anterior eye field (Figure 4A and Figure 5A). In *T. molitor*, the eyes consist of 440 regular facets, 50 ± 3.2 µm (N = 7) in diameter in both males and females; the cornea covers a surface area of 8.2 × 10^5^ µm^2^, for a density of facets of approximatively 537 n/mm^2^. The volume renderings and virtual sections (Figure 4C–E and Figure 6C) showed a corneal lens with a thickness of 38.7 ± 4.83 µm (N = 22) and an interocular distance of approximately 1.85 mm (Table 1). The area of crystalline cones is brighter (i.e., shows a higher attenuation) (Figure 6C), in contrast to the underneath layers (rhabdom) lined by the basal lamina, where a clear zone is not present. The axons converged towards the optic lobe (Figure 4D,E), which is divided in the lamina, medulla and lobula. The interommatidial angle was 6.89 ± 1.02° (N = 14). The facets in the eye of *T. castaneum* (Figure 5A) are 92 in both males and females. The volume renderings and virtual sections (Figure 5C–E) show a corneal lens with a thickness of 22.8 ± 3.07 µm (N = 10). The compound eye surface area reaches 9.0 × 10^4^ µm^2^, with a facet density of 1022 n/mm^2^, and the interocular distance reaches about 0.43 mm (Table 1). The area of the crystalline cones shows a higher level of attenuation than the rhabdom layer below, flats on the basal lamina. The axons converged towards the optic lobe connected to the cerebrum (Figure 5D,E). The lamina, medulla and lobula are also distinguishable. The interommatidial angle was 12.99 ± 1.2° (N = 9).

Comparing the 2D virtual sections of the compound eyes (Figure 6), two different structures can be distinguished. Indeed, both *P. melas* and *C. canaliculatum* (Figure 6A,B) show higher attenuation in the layer corresponding to the clear zone, interposed between the crystalline cones and the rhabdom layer. In contrast, in *T. molitor* and *T. castaneum* (Figure 6C,D), as no clear zone is present, the difference in attenuation between the different layers is not evident. Moreover, the eyes in tenebrionid beetles are characterised by the lowest facet density and the highest facet surface area (Table 1).

## 4. Discussion

The high resolution of the beetle virtual dissections obtained under SR-PhC micro-CT analyses was useful to observe the head in transversal, sagittal and frontal planes, and the 3D reconstructions have the advantage of facilitating the rotation of the sample on all axes. Moreover, the contrast between the different tissues allowed us to distinguish the complex internal structures inside the head capsule, moving within the 2D image stacks, or by cutting into the 3D models as rendered by the Drishti and Avizo software. Scanning (SEM) and transmission (TEM) electron microscopy analyses and histology have been largely used to study the external morphology and ultrastructure of insects [24,67,68], mainly to describe the sensorial equipment involved in detecting biotic and abiotic stimuli from environments [69,70,71,72,73,74], including the compound eyes [39,75,76]. However, these methods are limited for scanning the external surface, or require a high number of samples for ultrastructure and histological analyses [68]. Volume renderings of the compound eyes for each species analysed in our study provided adequate morphological information on the internal dioptric apparatus and sensorial parts with a low number of specimens. This is very useful for the study of vulnerable species such as *C. canaliculatum.* Moreover, the differences in attenuation obtained from the virtual sections allowed us to identify two basic types of compound eyes, according to whether or not the receptor layer and the dioptric apparatus appear separated, that characterise the superposition eyes of *P. melas* and the apposition structure of both *T. molitor* and *T. castaneum*. Although the analysed species differed in size, no differences were found in the quality of the resulting datasets in terms of detail visibility, confirming SR-PhC micro-CT as a useful tool to study the internal anatomy of miniature insects [5] such as *C. canaliculatum* and *T. castaneum,* as well as the nervous system and the optical lobe [77].

Our results also indicated that the suitable quality of the morphological data processed by SR-PhC micro-CT means the technique has high potential for application in ecological studies. The analysed models were four coleopteran species, which live in low light conditions, but with species-specific habitat demands. Variations in the structural characteristics of the compound eyes recorded in the studied species, such as the facet diameters, interommatidial angle and the number of ommatidia, were good indicators of the differences in behaviour, lifestyle and habitat preference. The superposition eyes, that lack pigment separating the cornea from rhabdomeres, are more sensitive to light because they permit all photoreceptors to use the corneal dioptric apparatus [24,76]. We found this structure in *P.* *m. italicus,* a generalist predator in the food web of agroecosystems [50], which is active over a broader intensity range and adjusts the sensitivity of its eyes to the different levels of environmental brightness. Facet density in *P.* *m. italicus* was found to be the highest among the described species, depending on the size and spacing of the ommatidia, and in accordance with the visual resolution requirements of a predatory lifestyle [78]. As tiny lenses are thought to deliver poor acuity because of diffraction, the high number of narrow-diameter facets increases light sensitivity and visual resolution in the visually challenging lifestyles of species such as *P.* *m. italicus,* which can be considered a visual hunter, according to previous studies on carabid beetles [27,28,29].

The external morphology of the eyes of *C. canaliculatum* is consistent with the 3D image of the orbital grooves shown in a previous study performed using SEM techniques [79]. Although the species has been indicated as anophthalmic [79], the SR-PhC micro-CT analyses revealed that the structure, previously considered to be non-functional because of the absence of facets, shows the typical sensorial area of a functioning superposition eye. However, the ommatidia are spread apart, occupying in total a lower surface area than that covered by the overlying cornea. *C. canaliculatum* is an obligate saproxylic species, inhabiting the rotten wood of mountain forests in central and southern Italy and Greece [47,48,80]. Thus, it probably needs a larger lens to increase the light incidence angle and achieves sufficient contrast sensitivity by increasing light transmittance crystalline cones in low light conditions [25,81]. We speculate that the cornea of the transparent cuticle increases the sensitivity of the eyes to photons for detecting the surrounding environment, as an adaptation to life in dim light conditions. Furthermore, *C. canaliculatum* shows the thickest lens in proportion to the head size among the described species, which requires further studies to clarify whether the eye is functional and to what extent.

*Tenebrio molitor* and *T. castaneum* live in food storage depots that occasionally offer a low illumination level. However, previous electrophysiological studies indicated that *T. molitor* is enabled to discriminate various wavelengths from visible to ultraviolet radiation [82,83,84]. Although there are no physiological or behavioural studies on the spectral sensitivity of *T. castaneum*, virtual sections and 3D renderings highlighted the typical structure of the light-adapted apposition eyes, which enable orientating at low light intensities in both the tenebrionid species. Moreover, *T. molitor* is among the described species, the one with the lowest facet density, and the ommatidium is indeed characterised by a larger surface in proportion to the head size.

In conclusion, this is the first study focusing on the use of SR-PhC micro-CT to describe the compound eye morphology in insects, and to our knowledge, this is also the first evidence of structured compound eyes in *C. canaliculatum*. Moreover, our results indicated that this is a useful non-destructive technique for investigating vulnerable, rare or difficult-to-collect species included on the IUCN red list—such as *C. canaliculatum*—affected by intensive forest management leading to deadwood reduction [48], and allows for additional analyses to be provided using low numbers of specimens. Some size-dependent limitations of structures were found for the reconstruction of smaller sensorial cells, such as the rhabdom reaching the cone, pigment and retinula cells. This method allows measurements of morphological parameters such as interocular distance, the density of facets, the thickness of the cornea and the number of ommatidia, which is useful in future interspecific comparative studies for understanding how different lifestyles and eye and brain morphology have co-evolved, under the selective pressure of biotic (food, predators) and abiotic (light) factors. Furthermore, conventional techniques adopted for the investigation of the eye, such as retinal dissection and histology, show several limitations in small specimens, such as *T. castaneum*, and are not applicable. In contrast, SR-PhC micro-CT allows morphological analysis by providing a high degree of detail, even in small species. However, our findings showed that the resolution and image quality of this technique make it a useful and reliable tool to describe the dioptric apparatus in situ and the general organization of the sensorial structure, without any deformation due to the manipulation requested for microscopic analyses.

## Figures and Tables

**Table 1:** Morphological parameters of studied species measured on 2D slices and volume renderings of beetles’ heads.

Species	N. of Ommatidia	Corneal Facet Surface Area (µm^2^)	Total Surface of Facets ^a^ (µm^2^)	Cornea Surface ^b^ (µm^2^)	Facet Density ^c^	Corneal Facet Lens Thickness (µm)	Interocular Distance (mm)	Head Size ^d^ (mm)
*Tribolium castaneum*	92	(1.02 ± 0.14) × 10^3^	(9.40 ± 1.31) × 10^4^	9.0 × 10^4^	1022	22.8 ± 0.97	0.43	0.7–0.7
*Tenebrio molitor*	440	(1.97 ± 0.26) × 10^3^	(8.67 ± 1.13) × 10^5^	8.2 × 10^5^	536.6	38.7 ± 1.03	1.85	2.68–2.8
*Pterostichus melas italicus*	1380	(0.50 ± 0.07) × 10^3^	(6.96 ± 0.99) × 10^5^	6.9 × 10^5^	2000	64.9 ± 1.68	2.73	3.39–3.45
*Clinidium canaliculatum*	70	(0.29 ± 0.06) × 10^3^	(2.09 ± 0.56) × 10^4^	4.5 × 10^4^	1555	50.94 ± 0.63	0.79	0.97–1.0

The values are expressed as mean ± standard deviation and the measured structures are named as indicated in Figure 1. ^a^ corneal facet surface area x number of ommatidia; ^b^ data from Avizo software; ^c^ number of ommatidia estimated/surface (mm^2^) of cornea; ^d^ length–width: measured from clypeus apex to neck base and between apices of eyes, respectively.

## Data Availability

Not applicable.
